# Supplementary material for: Phytochrome B and phytochrome-interacting-factor4 modulate tree seasonal growth in cold environments
Source: Nat Commun. 2025 Aug 30;16:8114. doi: 10.1038/s41467-025-63391-5 (PMC12398530; doi:10.1038/s41467-025-63391-5)
Supplement: Supplementary file 1 — Supplementary information [file 41467_2025_63391_MOESM1_ESM.pdf]

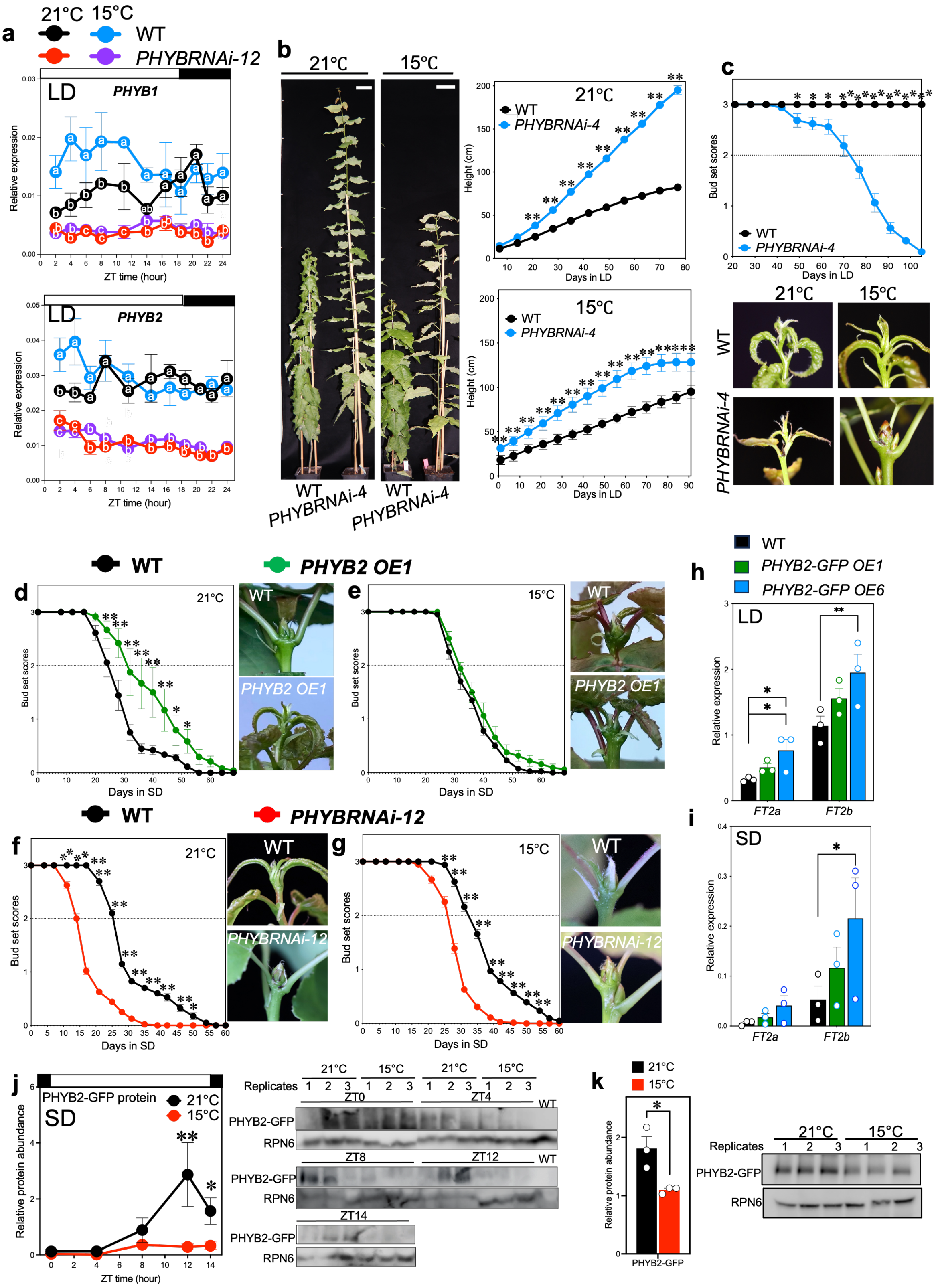

Supplementary Figure 1. ***PHYB*** **transgenic lines display altered responses to low temperatures.**

(a) Diurnal gene expression of *PHYB1* and *PHYB2* in leaves of WT and *PHYBRNAi-12* trees. (b) Height growth of WT and *PHYBRNAi-4* plants in LD. Representative pictures of trees are shown in the left panel. Bars indicate a length of 10 cm. (c) Bud set scores of WT and *PHYBRNAi-4* plants in LD. (d, e, f, g) Bud set scores of WT, *PHYB2 OE1* and *PHYBRNAi-12* plants in SD. Representative pictures of the shoot apices are shown in the lower panel (c) or the right panel(d-g). Pictures were taken when the bud set scores of plants started reaching stage 0.5.

The dotted lines marked bud set score 2, indicating the growth cessation stage. (h, i) Relative expression of *FT2a* and *FT2b* in leaves of WT and the two *PHYB2* overexpressing lines grown for eight weeks in LD and one week in SD. Samples were taken at ZT18. (j, k) Western blot analysis of PHYB2-GFP fusion protein in soil-grown (j) and *in vitro*-grown (k) *PHYB2-GFP OE6* plants under SD conditions at 21°C and 15°C. The blots were probed with an anti-GFP antibody to detect PHYB2-GFP and anti-RPN6 antibodies as a loading control. For soil-grown plants (j), leaf samples were collected at ZT0, 4, 8, 12, and 14, while *in vitro*-grown plants (k) were sampled at ZT8. The left panels display quantitative analysis of PHYB2-GFP protein levels from the western blots. Data are presented as mean ± SEM ($n = 3$ for a, b, h, i, k, j; $n = 8$ for c, e, f, g). Fisher's LSD post hoc tests were performed without multiple testing correction at all time points following ANOVA for all the datasets except dataset k, where a Student's t-test was performed instead. The statistically significant differences are indicated by non-matching letters in a ($p < 0.05$). Asterisks in b, c, d, f, g, h, i, j, and k indicate the levels of statistical significance, ✻ $p< 0.05$, ✻✻ $p< 0.01$. No statistical differences were detected in e. All experiments have been repeated at least two times with similar results.

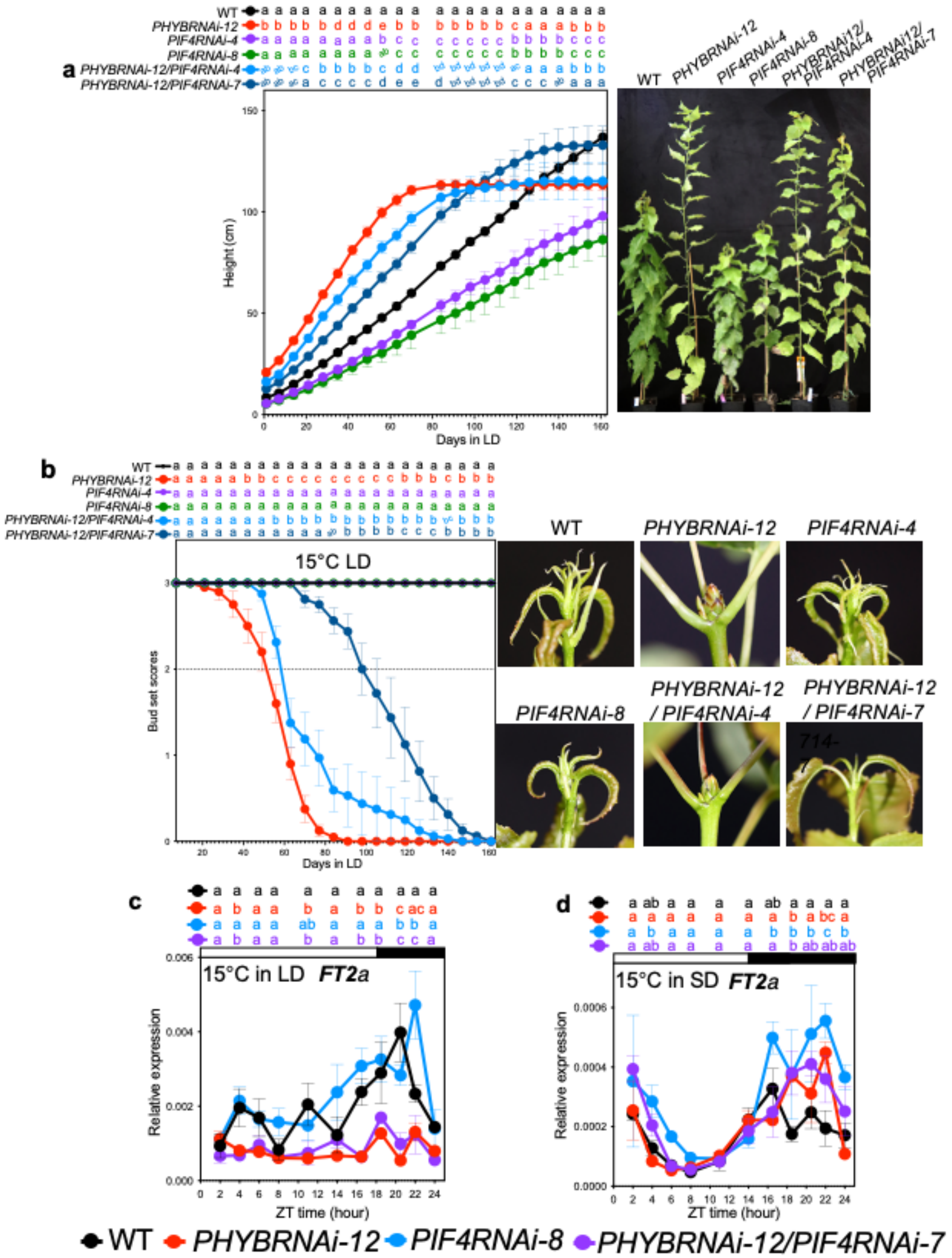

Supplementary Figure 2. *PHYB* and *PIF4* RNAi transgenic lines show that PIF4 specifically regulates growth at low temperatures.

(a) Height growth of WT, *PHYBRNAi-12*, *PIF4RNAi-4*, *PIF4RNAi-8*, *PHYBRNAi-12/PIF4RNAi-4* and *PHYBRNAi-12/PIF4RNAi-7* plants in 15°C LD. Representative pictures of whole trees are shown in the right panel. Bars indicate a length of 10 cm. Pictures were taken after 80 days in LD at 15 °C.(b) Bud set scores of WT, *PHYBRNAi-12*, *PIF4RNAi-4*, *PIF4RNAi-8*, *PHYBRNAi-12/ PIF4RNAi-4*, and *PHYBRNAi-12/ PIF4RNAi-7* plants in LD at 15°C. Representative pictures of the shoot apices are shown in the right panel. Pictures were taken when bud set scores of *PHYBRNAi-12* plants reached stage 0.5 (green closed apical bud). (c, d) Diurnal relative gene expression of *FT2a* in 15°C LD (c) and 15°C SD (d). Leaf samples were collected after 56 days in LD or 7 days in SD. Data are presented as mean ± SEM (n = 3). Following ANOVA, pairwise comparisons were performed using Fisher's LSD test without multiple testing correction at each time point. The statistically significant differences are indicated by non-matching letters ($p < 0.05$). All experiments have been repeated at least two times with similar results.

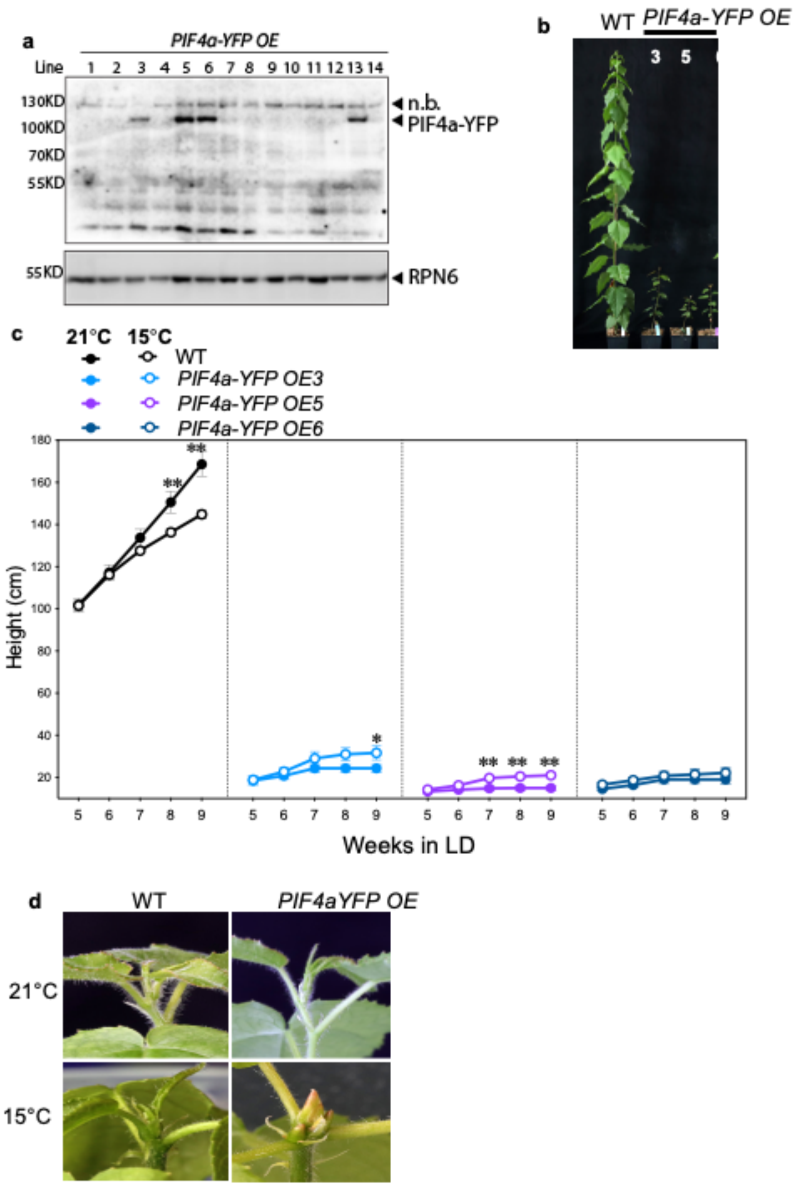

Supplementary Figure 3. ***PIF4a*** **overexpression suppresses aspen tree growth**

(a) Western blot analysis of *PIF4a-YFP OE3* transgenic lines using an anti-GFP antibody to detect PIF4a-YFP and an anti-RPN6 antibody as loading control. (b) Soil-grown plants of WT and three independent *PIF4a-YFP*-overexpressing lines. (c) Height growth of WT and three *PIF4a-YFP OE* lines in LD at 21°C and 15°C. Data are represented as mean ± SEM, n = 3. It has been repated two times with similar results.The asterisks represent significance levels (✻ p< 0.05, ✻✻ p< 0.01) based on Fisher's LSD test without multiple testing correction following ANOVA. (d) Shoot apices of WT and the *PIF4a-YFP OE3* plants grown in jars under 15°C or 21°C LD.

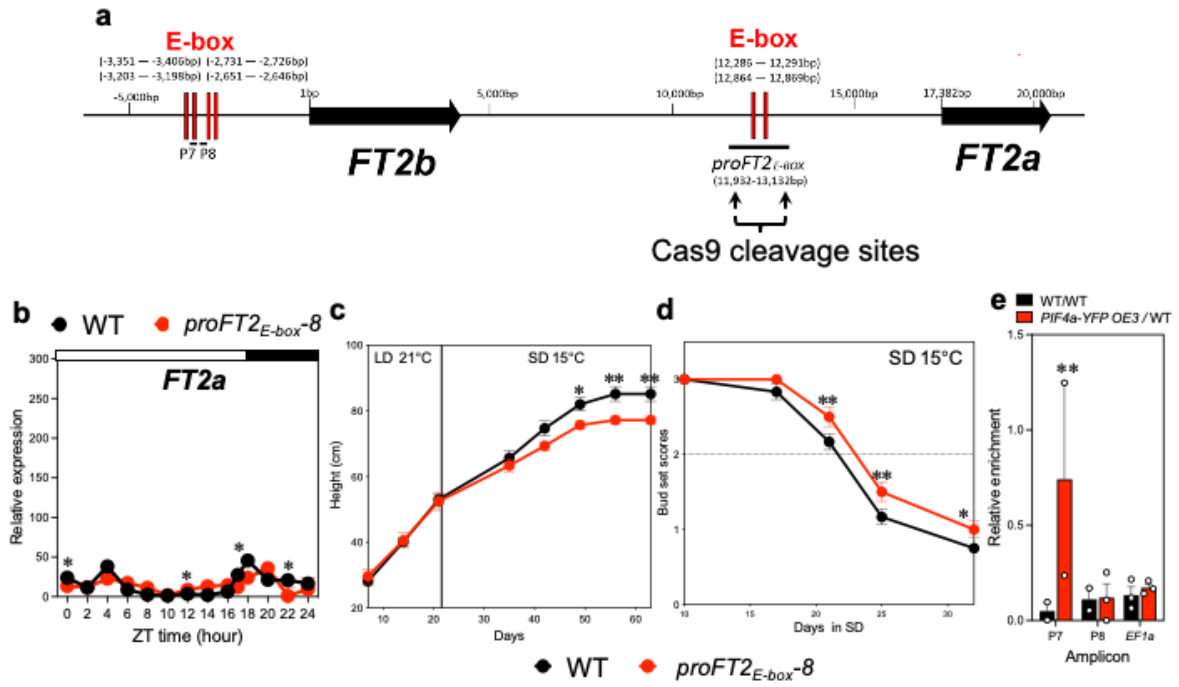

Supplementary Figure 4. E-box fragment-deleted plants show a delayed SD-induced growth cessation.

(a) Genomic organisation of the *FT2* paralogs in *Populus tremula*. The two arrows indicate the CRISPR/Cas9 cleavage sites. Black boxes indicate genomic regions of the two paralogs from the start to the stop codons. The red box shows the E-box (NACGTG, CACNTG) motifs and the potential binding sites for PIF4. (b) Diurnal relative expression of *FT2a* in leaves of the *proFT2E-box-8* mutant grown for 40 days in 21°C LD. (c) Height growth of WT and the *proFT2E-box-8* mutant. Plants were grown in LD at 21°C for 23 days, then shifted to SD at 15°C. (d) Bud set scores of WT and the *proFT2E-box-8* mutant during SD-induced growth cessation at 15°C. The dotted line marked bud set score 2 indicates the stage of growth cessation [26]. (e) Relative enrichment of upstream fragments of *FT2b* after CUT&Tag quantified by qPCR. Values are normalized against the input DNA before the enrichment. The unrelated elongation factor 1 alpha (*EF1a*, *Potra2n6c14161*) served as a negative control. Data are presented as mean ± SEM (n = 2 for b,e; n = 6 for c, d). Fisher's LSD post hoc tests (uncorrected for multiple comparisons) were performed at all time points following ANOVA. Asterisks in b,c,d indicate the levels of statistical significance, ✻ $p < 0.05$, ✻✻ $p < 0.01$. All experiments have been repeated at least two times with similar results.

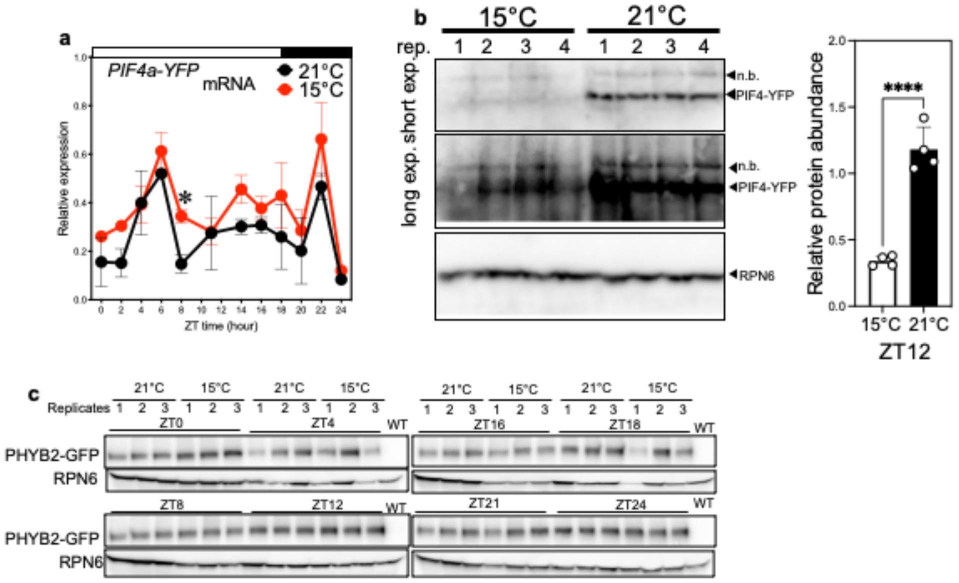

Supplementary Figure 5. Low temperature suppresses PIF4a protein accumulation in *in vitro*-grown plants.

(a) Diurnal relative transcriptional levels of the *PIF4a-YFP* transgene in *PIF4A-YFP OE3* leaves at 21°C and 15°C LD. Data are represented as mean ± SEM, n = 3. Asterisks indicate the levels of statistical significance determined by two-way ANOVA Fisher's test, ✻ $p < 0.05$. (b) Western blot analysis of *PIF4a-YFP OE3* plants grown in jars, using an anti-GFP antibody to detect PIF4a-YFP and anti-RPN6 antibody as loading controls. PIF4a-YFP indicates the fusion proteins detected in each biological replicate, and n.b. indicates a non-specific band. The graph on the right shows the quantification of the western blot. Data are represented as mean ± SEM, n = 4. Asterisks indicate a statistically significant difference determined by a Student's t-test, ✻✻✻✻ $p < 0.0001$. (c) Western blot analysis of PHYB2-GFP fusion protein in *PHYB2-GFP OE6* soil-grown plants under LD conditions at 21°C and 15°C. Leaf samples were collected at ZT0, 4, 8, 12, 16, 18, 21, and 24. Quantifications are shown in Figure 5f. All experiments have been repeated two times with similar results.

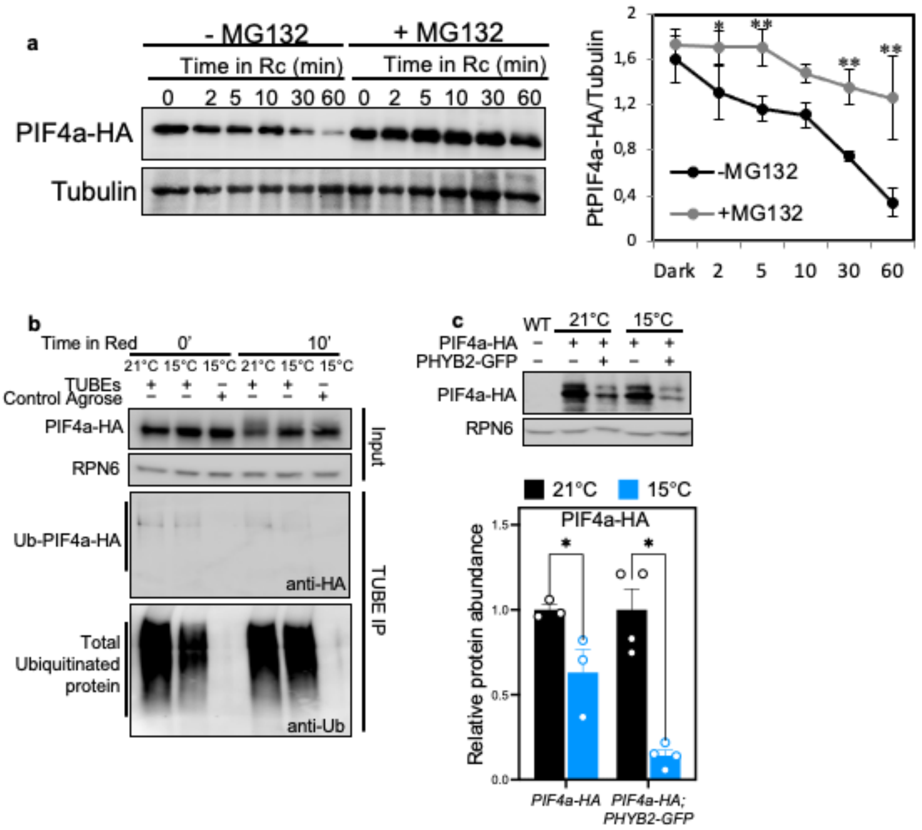

Supplementary Figure 6. Low temperature promotes PIF4 degradation.

(a) PIF4a-HA undergoes proteasome-mediated protein degradation triggered by red light in aspen trees. Western blot of PIF4a-HA fusion protein in *PIF4a-HA* overexpressing plants in response to red light with or without MG132 treatment, using an anti-HA antibody to detect PIF4a-HA and an anti-tubulin antibody as loading control. The right panel shows the quantification of PIF4a-HA fusion protein by western blot. Data are represented as mean ± SEM, $n = 2$. (b) TUBEs assays of ubiquitinated proteins in *PIF4a-HA* overexpressing plants at 21°C and 15°C post 0 and 10 minutes of red light treatment. Total ubiquitinated proteins were immunoprecipitated with agarose-TUBE2, then analyzed by western blotting with anti-HA antibodies for detection of PIF4-HA and anti-ubiquitin antibodies as loading controls. Control agarose that were not TUBEs-conjugated were used as negative controls. Anti-RPN6 antibodies were used as loading controls for input samples. (c) Western blot of PIF4a-HA fusion protein in *35S::PIF4a-HA-1* and *35S::PIF4a-HA-1/35S::PHYB2-GFP* trees at two temperatures. Leaf samples were taken at ZT8 of 6-week-old LD grown trees. The bottom panel shows the quantification of PIF4a-HA fusion protein by western blot. Data were normalised by the 21°C samples and represented as mean ± SEM, $n = 3$. Fisher's LSD post hoc tests (uncorrected for multiple comparisons) were performed following ANOVA at all time points (a) and at the two temperatures (c). Asterisks indicate the levels of statistical significance, ✻ $p < 0.05$, ✻✻ $p < 0.01$. All experiments have been repeated two times with similar results.

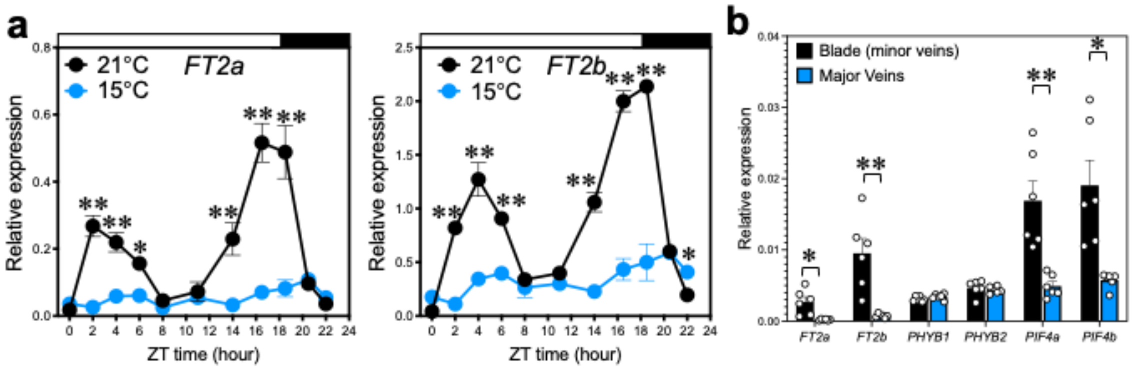

Supplementary Figure 7. Gene expression analysis of *FT2, PHYB*, and *PIF4*
(a)Diurnal relative gene expression of *FT2a* and *FT2b* in leaves of WT under LD conditions at 21°C or 15°C. (b) Gene expression of *FT2a*, *FT2b*, *PHYB1*, *PHYB2*, *PIF4a*, and *PIF4b* in major veins and leaf blades (minor veins). Data are presented as mean ± SEM (n = 3 for a; n = 6 for b). Fisher's LSD post hoc tests were performed at all time points following ANOVA. Asterisks indicate the levels of statistical significance, ✻ $p< 0.05$, ✻✻ $p< 0.01$. All experiments have been repeated at least two times with similar results.

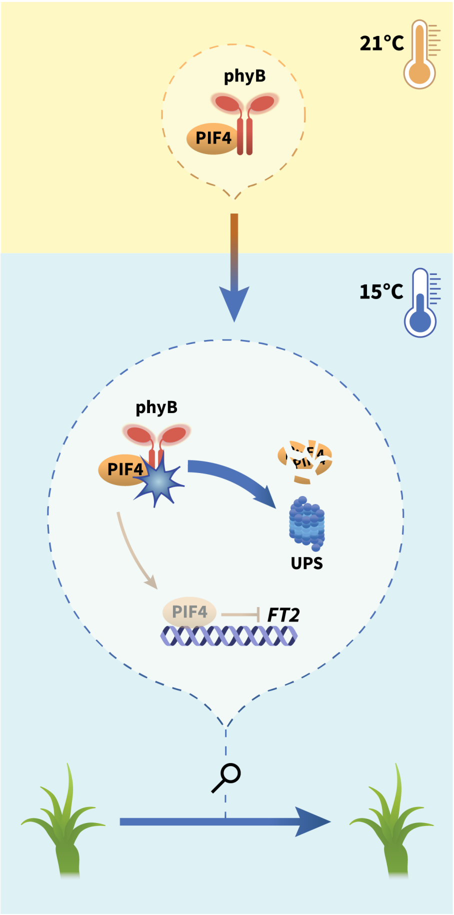

Supplementary Figure 8. Model for the role of the phyB-PIF4 module in promoting growth at low temperatures.
PIF4 binds to a negative regulatory element in the genomic region of *FT2* to suppress its transcription. phyB interacts with PIF4 and targets this protein for proteasome-mediated degradation. When the temperature drops from 21°C to 15°C, the interaction between phyB and PIF4 is enhanced to promote more PIF4 degradation, which prevents aspen trees from premature growth cessation under otherwise permissive LD conditions.

**qPCR primers**	**Forward**	**Revers**
YLS8	ACTGTTTTCTTCTTCAACGCTC	TGAAAAGCACCAACCCATTTAG
eIF5A	CAACCAACATGTCGGACGAG	CCAGCTTGCTGAGGGTATGTCTT
FT2a	AGCCCAAGGCCTACAGCAGGAA	GGGAATCTTTCTCTCATGAT
FT2b	AGCCCAAGGCCGACAGCGGGAA	GGGAATCTTTCTCTCACGAC
PIF4a	ATTGAGACCGGTACAGGCTTG	GGCTGGCTTGTTTTCCACTGC
PIF4a-YFP	CAGATAAAGCATCAATGTTA	ACACGCTGAACTTGTGGCCGTTTA
**ChIP-qPCR primers**	**Forward**	**Revers**
P1	GAGTGGAATTACAAGAACAG	CACAGAAGTTGATGTATACG
P2	GCTTCGTATACATCAACTTC	CACTTCGCAGCTTGTATTCA
P3	CCTCTTAACCAGTAACCTA	CAAGAGGGTCAATATGAGT
P4	CCTTATCCCAAGTTAATCCT	TGGTTAATGTCAATGCAAGG
P5	CCATATATCTTCGAGCGTTGCA	AAGCTGGGTTCGAGTAAAG
P6	GTATGCCGAGATGGAGACT	TCATAAAGCATGCATGGACC
P7	TCGGAGTGCATTTATTGGAG	TGGAGTGAGCAAGTATGAC
P8	ACGCACATATCAACTCTTTG	CCCTGTAACCAAGCAATTAT
EF1a	GGCAAGGAGAAGGTACACAT	CAATCACACGCTTGTCAATA
**sgRNA**		
proFT2-E-box-sg-1	GTACGCAAATGCTACGTGCA	
proFT2-E-box-sg-2	TATTGACCCGAGCTTTGTCA	
**Y1H primer**		
FT2a-1st PER-attB4F	GGGACAACTTTGTATAGAAAAGTTGAATCTAAGCGTGTTGTTGTA	
FT2a-1st PER-attB1R	GGGGACTGCTTTTTTGTACAAACTTGTCTAAAAAACAACTCGGGAGT	
1H1FW	GTTCGGAGATTACCGAATCAA	
HIS293RV	GGGACCACCCTTTAAAGAGA	
LacZ592RV	ATGCGCTCAGGTCAAATTCAGA	
M13F	GTAAAACGACGGCCAGT	
***FT2b* amplicon sequencing primers**		
FT2b-forward	AGCCCAAGGCCGACAGCGGGAA	
FT2b-reverse	AAAATGAGTGGACAAAATTGCGAC	

Supplementary Table 1: List of used sequences

Transgenic line	Allele 1 editing	Allele 2 editing	
		sgRNA-1	sgRNA-2
proFT2$_{E\text{-}box}$-8	1253 bp fragment deletion	GTACGCAAATGCTACGT–GCA<br>GTACGCAAATGCTACGTTGCA	TATTGACCCGAGCTTTGTCA<br>TATTGACCCGAGCTTTGTCA
proFT2$_{E\text{-}box}$-13	1253 bp fragment deletion	GTACGCAAATGCTACGT–GCA<br>GTACGCAAATGCTACGTTGCA	TATTGACCCGAGCTTTGTCA<br>TATTGACCCGAGCTTTGTCA
proFT2$_{E\text{-}box}$-25	1253 bp fragment deletion	GTACGCAAATGCTACGTGCA<br>GTACGCAAATGCTACGTGCA	TATTGACCCGAGCTTTGTCA<br>TATTGACCCGAGCTTTGTCA

Supplementary Table 2: The *proFT2$_{E\text{-}box}$* CRISPR lines screening.
The red letters indicate the Indel mutations.
